# Assessing Financial Impacts of Subclinical Mastitis on Colombian Dairy Farms

**DOI:** 10.3389/fvets.2018.00273

**Published:** 2018-11-27

**Authors:** Jaime Romero, Efraín Benavides, Carlos Meza

**Affiliations:** ^1^Inter-American Institute for Cooperation on Agriculture, Lima, Peru; ^2^Faculty of Agricultural Sciences, Universidad de La Salle, Bogota, Colombia; ^3^Faculty of Economic and Social Sciences, Universidad de La Salle, Bogota, Colombia

**Keywords:** animal health economics, bovine mastitis, producers attitudes, production system, veterinary epidemiology

## Abstract

Bovine mastitis is a dairy cattle disease with high economic impact. Subclinical mastitis (SCM) contributes to most of the financial losses. Colombia dairy sector accounts for 2.3% of the gross domestic product (GDP) and 24.3% of the livestock GDP. Milk production reaches 6,500 million liters/year from nearly 500,000 cattle farms and is mainly based on small-scale production systems. This study evaluates the financial impact of SCM and the potential for its control in three dairy farm strata in a region in Colombia. The objectives of the study were 1) to determine the perception of farmers about the SCM problem on their farms, 2) to assess prevalence and financial impact of SCM on farms and in the “Area five” sanitary region of the Bogota plateau, and 3) to assess costs and effectiveness of control methods of SCM. Information about disease management and decision-making process was obtained through a participatory epidemiology workshop and applying a semi-structured survey. A two-stage stratified cross sectional epidemiological study was conducted on dairy cattle from a region with approximately 400 farms and 12,000 cows, with a sample size of 55 farms. Prevalence of SCM was calculated by defining a cow as positive for the disease when any quarter had a somatic cell count (SCC) higher than 250 × 10^3^ cells/ml. The prevalence of SCM in cows was 55.2%; significant differences were found between strata. Assessment of the financial impact of SCM in terms of milk losses was conducted using spreadsheet models. Milk production losses per farm ranged from 1.3% to 13.5%, and the economic impact in the region was estimated over USD $800.000 per year. The financial impact was greater in small- and medium-sized farms than large farms, and it was associated with the severity of SCC per quarter. Principal component analysis showed interactions, irrespective of the individual effect, and suggested three main groups of control interventions: application of basic milking hygiene practices, increase in the level of hygiene practices and veterinary advice, and SCM diagnosis and dry-cow treatment. Lack of information on management and production at farms promotes intuitive decision-making. Further research for the deeper understanding of intervention costs and effectiveness is suggested.

## Introduction

Bovine mastitis is a disease with a high prevalence in dairy cattle worldwide with a major impact owing to economic losses caused at various levels of the dairy value chain (1–3). Mastitis is classified as clinical or subclinical depending on the visibility of effects of inflammation of the mammary gland. Subclinical mastitis (SCM) does not produce visible effects on udder or milk quality (4, 5) but has important effects on milk composition, mainly an increase in SCC (5, 6).

Studies to determine the economic impact of bovine mastitis have been conducted mainly in developed countries (1, 2, 7). Mastitis losses are due to reduced milk production, cost of treatments, and culling, accounting for 78%, 8%, and 14%, respectively (7). However, the economic impact of mastitis varies and should be calculated at the farm or herd level and depends on local, regional, epidemiological, managerial, and economic conditions (2, 3, 7, 8). Most losses are associated with SCM, defined as an increase in the content of SCC in milk, which many producers undervalue, owing to the lack of visible abnormalities in milk, which requires specific detection methods such as the California Mastitis Test (CMT) (4, 6). Additional disease losses are generated from disease management to the presence of both clinical and subclinical mastitis at farm (8–11).

The Food and Agriculture Organization (FAO) highlighted the importance of providing information on the economic dimension of the disease in resource-poor environments (12). In relation to SCM, FAO states that this hidden disease needs to be recognized early by producers, since its effective management does not depend solely on a simple recommendation but instead on multiple recommendations based on a better understanding of the disease.

In developing countries, the economic impact of SCM in small- and medium-sized farms varies according to the level of milk production per cow and the intensity of the production systems. In Costa Rica, milk production losses per cow with SCM were estimated at 1.6 kg day^−1^ for daily milk yield (4). In Ethiopian crossbreed dairy systems, milk production was reduced by 1.2%, 6.3%, and 33%, respectively, in quarters with CMT scores 1+, 2+, and 3+ (11). In smallholder dairy farms in Tanzania, with a prevalence of SCM of 46.2%, intra mammary antibiotics significantly reduced the proportion of bacteriologically positive quarters in the short-term (14 days post-infusion), but teat dipping had no detectable effect on bacteriological infection and CMT positive quarters (5).

In Colombia, total milk production is approximately 6,500 million tons/year, produced in dual-purpose systems (4.8 million cows) and specialized dairies (600,000 cows), with the latter mainly based on the Holstein breed. Less than half of the total produced milk (approximately 3,200 million tons/year) comes from the formal milk processing industry, including pasteurization. Milk is produced in small-scale production herds, 395,000 cattle producers, which represent 80% of the cattle producers in the country (13). The dairy sector in the country accounts for 2.3% of the gross domestic product (GDP) and 24.3% of the country's livestock GDP, generating nearly 717,434 direct employments (14). In Colombia, there are legislation and policies about price incentives for raw milk quality according to total solid and bacterial contents (CFU, colony forming units), but there are neither penalties nor economic incentives with low or high somatic cell count (SCC) in milk. Some pasteurization plants pay incentives for low SCC in bulk tank milk.

Previous studies on bovine mastitis in Colombia focused on the microbiological side of the problem, using CMT as the diagnostic tool and bacteriological culture to confirm the identity of the pathogen. In a longitudinal study of ten herds in the Bogota Plateau, 47% of the cows presented SCM (25% of quarters), and the predominant bacterium was *Streptococcus agalactiae* (15). In small-scale production systems in eastern Antioquia, 12.3% of the quarters were positive, *S. agalactiae* being the most frequently isolated organism (16). A more recent study of intensive production systems at the Bogota Plateau found 34% of the quarter to be positive for SCM, with 29% of the isolates being *Staphylococcus aureus*, while *Streptococcus agalactiae* was isolated in 6.8% of the samples (17).

During 2014–2016, the University of La Salle and FEDEGAN (National Federation of Livestock owners) executed a research project that aimed to generate epidemiological information on mastitis and determine the economic impact of bovine mastitis on farms located in the Bogota Plateau. The overall objective of the study was to provide epidemiologically based information on the importance and impact of SCM on farms in the municipality of Zipaquira, determining the behavior and perception of producers regarding the control and prevention of the disease, to establish the potential benefit of control alternatives and improve decision-making in that matter. This paper presents results of the financial assessment of the impact of SCM at a farm level in the region and their relationships with farm practices. Thus, the objectives of the study were 1) to determine the perception of farmers about SCM on their farms, 2) to assess the financial impacts of SCM on farms with different sizes, and 3) to assess costs and effectiveness of different control methods for SCM.

## Materials and methods

### Description of study region

This study was based on the field data collected during the research project entitled “Epidemiological and economic components as a basis for decision-making in the control of bovine mastitis in cattle farms in Zipaquira (Cundinamarca).” The project was funded by the University of La Salle and FEDEGAN, in collaboration with the committee of livestock producers of “Area Five” and the Inter-American Institute for Cooperation on Agriculture (IICA). The sanitary “Area Five” for foot and mouth disease vaccination encompasses 13 municipalities centered in Zipaquira in the Bogota Plateau at an altitude of 2,650 meters above sea level (m.a.s.l). Cattle population in the Bogota Plateau is about 140,000 cattle and 7,751 farms, while the Area Five (study area) population contains 16,598 cattle and 365 farms (Table 1).

**Table 1:** Cross sectional survey, sampling procedure (assumes 95% CI and 10% accepted error) based on the population of the study area.

**Farm strata**	**Number of cows by farm in the stratum**	**Number of Farms**	**Number of bovine heads**	**Number of cows**	**Average cows per farm**	**Number of sampled farms**	**Number of sampled cows per farm**	**Number of sampled cows per stratum**
Small	10–25	188	3,101	2,171	12	28	9	230
Medium	26–100	139	6,717	4,702	34	21	18	372
Large	>100	38	6,780	4,746	125	6	28	168
Total		365	16,598	11,619	–	55	–	770

### Data collection

Data were collected in a participatory workshop in Area Five and through both a cross-sectional epidemiological study of the prevalence of SCM and a questionnaire survey among producers of mastitis management practices (18–21).

### Participatory epidemiology workshop

The Area Five committee invited regional cattle producers with a total participation of 55 producers. During the workshop, they were asked to answer two questions: First, if mastitis is a problem, please describe what do you think are the main effects of this condition? Second, what actions do you implement to prevent and control mastitis in your farm? Producers wrote up their answers on cards, using one card per answer. Cards were posted on a wall in order to be discussed among participants. Cards were kept, and results were discussed at the workshop with the participants (18).

### Cross-sectional study

**Figure 1:**
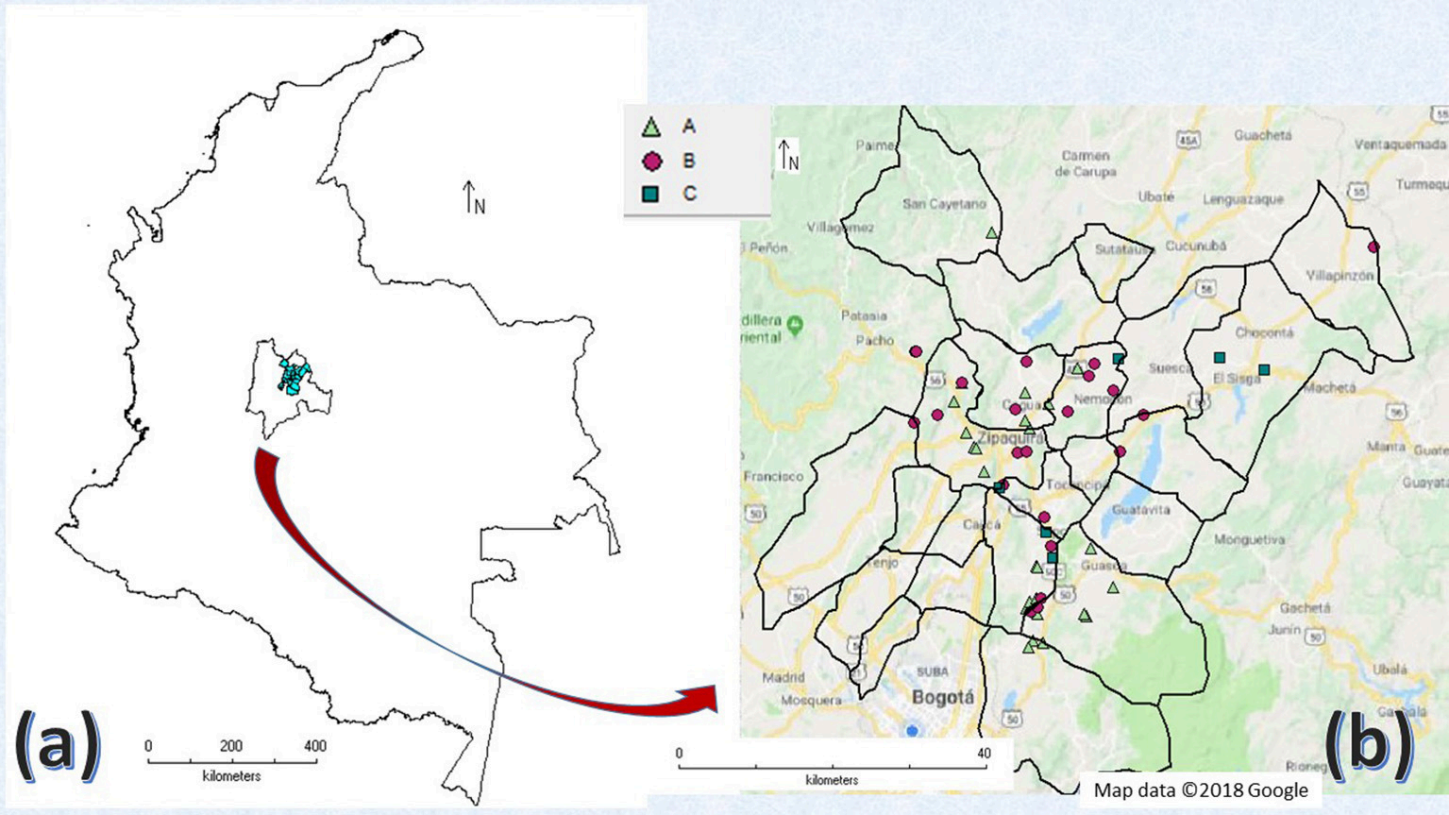
**(a)** Area of survey location: Area Five of the Bogota plateau at Colombia and Cundinamarca department. **(b)** Farm locations at survey area discriminated by strata: A, Small; B, Medium; C, Large. (Maps were prepared using the DIVA-GIS software and geographic images from Google maps).

A prevalence study of SCM was conducted in the area during the first semester of 2015. Farms were stratified according to the size of the production system (number of animals per farm) based on the number of farms in the study area registered by FEDEGAN (Table 1). Sample size of farms in each stratum was calculated by applying a probabilistic model for the estimation of frequencies (22), using the WinEpi software^1^, assuming the presence of SCM in 80% of the farms, as shown in previous studies in the country (15–17), an accepted error of 10%, and a confidence level of 95%. Therefore, a sample of 55 farms was established and assigned to the strata according to the sampling fraction and the number of farms per strata (Table 1). In addition, farms were selected based on the producer's willingness to participate in the study. Figure 1 shows the geographical location of the sampled farms in the study area.

The cattle sample size per farm was established using a probabilistic model, assuming SCM prevalence of 10% within the farm, an accepted error of 10%, and a confidence level of 95%. Table 1 indicates the number of sampled animals at each farm by stratum. At herd level, sampled cows were selected at random from milking cow lists.

From each cow, an aseptic sample of milk was collected from each quarter at the milking parlor in the morning (23). Each milk sample was analyzed for SCC using the Porta SCC^Ⓡ^ system (24). Individual quarter samples showing an SCC higher than 250 × 10^3^ cells/ml were considered positive for SCM. In addition, a cow was considered as “positive” if it had at least one positive quarter. A sample was considered as having “high SCC” when the count exceeded 1,000 x 10^3^ cells/ml. In the results, each quarter was categorized as negative (<250 × 10^3^ cells/ml), or positive to SCM: low SCC (between 250–1,000 × 10^3^ cells/ml), or high SCC (>1,000 × 10^3^ cells). Additionally, the lost quarters were counted and registered. Positive samples were cultured in blood agar and MacConkey medium, following the protocol of Sears and McCartie (23). A bulk tank sample was also collected at each farm, and SCC and bacteriological analysis were conducted.

The protocol of the study was approved by the Ethics Committee of the program of Veterinary Medicine of the Faculty of Agricultural Sciences of Universidad de La Salle, Bogota and the Research Vice-rectory of Universidad de La Salle. As part of the study protocol, the producers signed an informed consent.

### Questionnaire survey

A questionnaire survey was conducted in twice the number of farms initially required in the cross sectional study, having sufficient number of producers interviewed. Questionnaires were completed at all farms intended to participate in the cross sectional study, and additional questionnaires were filled out from neighboring farms and producers attending an animal live market in the region. Farms to be included were selected based on convenience and willingness to cooperate. A total of 103 questionnaires were completed, corresponding to 28% of the total farms within the study region.

The questionnaire included 80 questions divided in nine sections as follows: (a) respondent information; (b) farm general data; (c) clinical mastitis management; (d) SCM management; (e) milk production; (f) hand milking procedures; (g) mechanical milking system; (h) dry cow management; and (i) additional observations. The questionnaire was completed by an interviewer based on the answers from the owner of the farm or the person responsible for making decisions related to the milking process at the farm level. Only a fraction of the collected information was used in this study.

### Data analysis and spreadsheet modeling

Descriptive statistics were performed, using the Excel © (25) spreadsheet, to qualitatively sum up producer attitude and perception toward mastitis prevention and control expressed during the participatory workshop (55 participants) and questionnaire farm survey (103 participants).

From the cross-sectional study, the prevalence of SCM was calculated per farm as the number animals positive for SCM divided by total sampled animals. The confidence interval (CI) per farm was calculated using the disease measurement module of WinEpi (for calculation of prevalence from a sample), using a known population size and a 95% confidence level, taking into account positive animals to SCM, total sampled animals, and total number of cows present in each farm. For the calculation of prevalence and CI at the strata and regional levels, data was processed using the two-stage prevalence survey analysis tool of Ausvet epitools^Ⓡ^^2^. Following the same approach, the prevalence of SCM in cows at the strata and regional levels was estimated by accounting for positive animals, sampled animals, and total population from FEDEGAN's records to strata and region. Differences in prevalence by strata were established by using the chi square test (25). Finally, the average farm prevalence per stratum and the range of values were calculated from individual farm prevalence calculations. The prevalence of farms having at least one cow with SCM was calculated from the sample size of farms and the total number of farms by strata.

Proportions of lost quarters and SCM positive quarters (both low and high SCC quarters) were calculated per farm using cross-sectional study results.

The financial impact of SCM was assessed by focusing on milk losses as the main source of direct cost (8, 26, 27). Milk losses per farm resulted in differences between daily potential milk production and reported daily production.

The potential daily production of milk per farm was estimated using the data from the cross-sectional study and a model based on a spreadsheet. From the average daily milk production recorded in the cross-sectional study for each farm, the increase in the potential production of milk was calculated, simulating the production of milk that would be reached if there were no cases of mastitis or quarters lost. The figures of losses associated with the results of the SCC test per quarter were adapted from Mungube et al. (11) and used as follows: reduction of 2% in quarters with > 250,000 cells/ml, reduction of 33% in quarters with > 1,000,000 cells/ml, and reduction of 100% in lost quarters.

The model allows the estimation of milk losses and their financial value per farm (using local milk prices at the farm level). Subsequently, the individual results were adjusted to 10 cows and a lactation duration of 305 days per year. The spreadsheet model used the following equations:

$$DPMQ={DRMQ}^{*}\left(TQ/\left(TQ-UQE\right)\right),$$

where

*DPMQ* = daily potential milk production per quarter

*DRMQ* = daily recorded milk production per quarter

*TQ* = total quarters

*UQE* = unproductive quarter equivalence

The *DRMQ* was calculated per farm from the recorded average daily production per cow divided by four.

In addition, *TQ* was calculated multiplying the total milking cows per farm by four.

$$\text{UQE}={\text{TQ}}^{*}\left(\left(1^{*}\text{PLQ}\right)+\left(0.02^{*}\text{PLSQ}\right)+\left(0.33^{*}\text{PHSQ}\right)\right),$$

where

*PLQ* = prevalence of lost quarters per farm

*PLSQ* = : prevalence of low SCC quarters per farm (between 250–1,000 × 10^3^ cells/ml)

*PHSQ* = prevalence of high SCC quarters per farm (>1,000 × 10^3^ cells/ml).

The yearly milk losses per farm were calculated using the results from the cross-sectional study and adjusted to lactation length of 305 days per milking cow year, following these equations:

$$DML=DPMQ^{*}UQE,$$

where

*DML* = daily milk losses per farm.

Therefore, the model allows the estimation of the effect of SCM milk losses.

% Milk losses per farm =YML/YPM,

where

YML (yearly milk losses per farm) = DML^*^305

*YPM* = yearly potential milk production

*YPM* = (DPMQ^*^4)^*^milking cows ^*^305

Finally, the USD value of milk losses was calculated per farm using the reported price at farm. The exchange rate of $2,912 Colombian pesos per dollar was used as the official exchange rate on the date of the survey.

In order to reduce the effect of herd size on the absolute yearly milk losses, both yearly milk production losses and values were adjusted to 10 cows/year per farm using the following equation:

$$\text{A10CML}={\left(\text{YML}/\text{milking cows}\right)}^{*}10,$$

where

*A10CML* = adjusted milk losses 10 cows/year

Value of *A10CML* = farm milk price ^*^ *A10CML*

Descriptive statistics (mean, minimal, and maximal values and standard error) were built for the whole study and per strata. Statistical significance of mean differences per stratum was analyzed using one-way analysis of variance (25) for both adjusted 10 cow year and farm absolute milk losses and values.

Regional losses were estimated from total *YPM, YML*, and its monetary value per stratum and scaling up to the region using the sampled farm proportion from the regional total, using the data from FEDEGAN statistics (Table 1), and regional total amounts were the added result of the strata.

The costs of the most frequent preventive measures were estimated based on the results of the questionnaire survey (n: 103) about control measures and using field market prices of both input and labor. Regional expenditure was estimated using frequency of answers at the survey and the standardized herd size with 10 cows.

In order to infer the effect of control measures on SCM and losses, an ANOVA regression model was run (25). The independent variables came from the qualitative data from the cross sectional farm questionnaire survey (n: 55) about preventive and control measures (**Table 3**), and the value of *A10CML* was the dependent variable. Variance, inflation factors, and White and Breusch-Godfrey tests were applied for multicollinearity, heteroskedasticity, and auto correlation. Based on these test results and owing to the multicollinearity and heteroskedasticity found in the ANOVA model, a principal component analysis (PCA) was applied to the control measures (**Table 3**) in order to reduce both the number of variables to be analyzed and the variance.

Data appropriateness for the PCA was examined through the Kaiser-Meyer-Olkin (KMO) and Bartlett's test (28). Standard procedure for PCA was followed (28), starting with the identification of eigenvalues for each component, and followed by the extraction and the rotation of these eigenvalues. Finally, the proportional contribution of the variance of the data set was determined.

The proportion of the variance was defined as a linear estimate of the following form.

$$\begin{array}{l} An=\alpha_{n}X_{i}+\beta_{n}\overset{k}{\underset{i=1}{\sum }}Y_{i}+\delta_{n}Z_{i}+\mu \\ Bn=\alpha_{n}X_{i}+\beta_{n}\overset{k}{\underset{i=1}{\sum }}Y_{i}+\delta_{n}Z_{i}+\mu \\ Cn=\alpha_{n}X_{i}+\beta_{n}\overset{k}{\underset{i=1}{\sum }}Y_{i}+\delta_{n}Z_{i}+\mu , \end{array}$$

where

*A*_*n*_*, B*_*n*_, and *C*_*n*_ correspond to the farms in each stratum (A = small, B = medium, C = large),

α_*n*_, β_*n*_, and δ_*n*_ correspond to the coefficients for each of the independent variables of the model, namely:

X_i_ corresponds to the use or non-use of the CMT test on the farm; Y_i_ relates to the combination of the use of routine milking practices; Z_i_ describes the existence or inexistence of veterinary services, for each of the farms; and μ is the estimation error that includes the variables that were not included in the model.

Communalities or contribution to the variance of the data set were established following Kaiser's rule (variance over 1.0), and principal factors were established (28). Afterwards, a linear regression model (LRM) was run with the principal factors as independent variables and the value of *A10CML* as a dependent variable. Similarly, the LRM was tested with the White test for heteroskedasticity and the Breusch-Godfrey serial correlation LM test.

## Results

### Prevalence of SCM and the associated milk production losses

The cross-sectional study demonstrated that the overall individual prevalence of SCM in cows of Area Five in the Bogota Plateau was 55.2% (CI 95% = 43.1–67.3%; within farm variance = 0.195; between farm variance = 0.038). Table 2 shows the prevalence of SCM in cows according to the farm stratum. There were differences between strata in the estimated values of the prevalence of SCM in cows using values for total cows in the region (*X*^2^ = 1399.6; *p* < 0.0001). The quarter prevalence of SCM was 27.8%, 40.4%, and 14.7% for small, medium, and large farms, respectively. The proportions were different between strata (*X*^2^ = 146.68; *p* < 0.0001). At the farm level, only one out of the 55 farms had no cows with SCM; this was a small farm (19).

**Table 2:** Prevalence of SCM and milk losses in farms per stratum in the Bogota plateau.

**Strata (N° cows)**	**Stratum % prevalence of SCM^♦^ (95% CI)**	**Average cow SCM prevalence of farms by stratum (range)**	**Average milk loss due to SCM (%)**	**% Milk loss due to SCM (range)**
Small (10–25)	55.6 (41.5–69.7)	55.5 (0–100)	3.97	0–12.80
Medium (26–100)	74.3 (62.8–85.8)	42.5 (22.5–64.3)	6.14	1.26–13.53
Large (>100)	36.0 (27.5–44.5)	35.1 (25.0–50.0)	3.57	1.69–5.53

♦ *Correspond to cows having a quarter with SCC > 250 × 10^3^ cells /ml*.

*Stratum prevalence calculated using the two stage prevalence module of Epitools (SRS-stratified), using information of total population of sampled farms and total numbers of farms and animals by strata*.

Prevalence of SCM was calculated for each of the farms sampled in the study, and the average value and range by stratum is presented in the third column of Table 2. In addition, CI 95% for each farm was also calculated (data not shown).

Table 2 shows the average of percentage of estimated milk production losses and its range per stratum. In small farms, the percentage of estimated milk production losses due to SCM per farm fluctuated from 0% to 12.8%, but a higher percentage of losses was observed in the medium sized farms, ranging from 1.3% to 13.5%. High dispersion of percentage of losses among farms was found irrespective of the stratum, while the scattering of values was less extended in the large farms.

### Disease perception and control prevention measures

**Figure 2:**
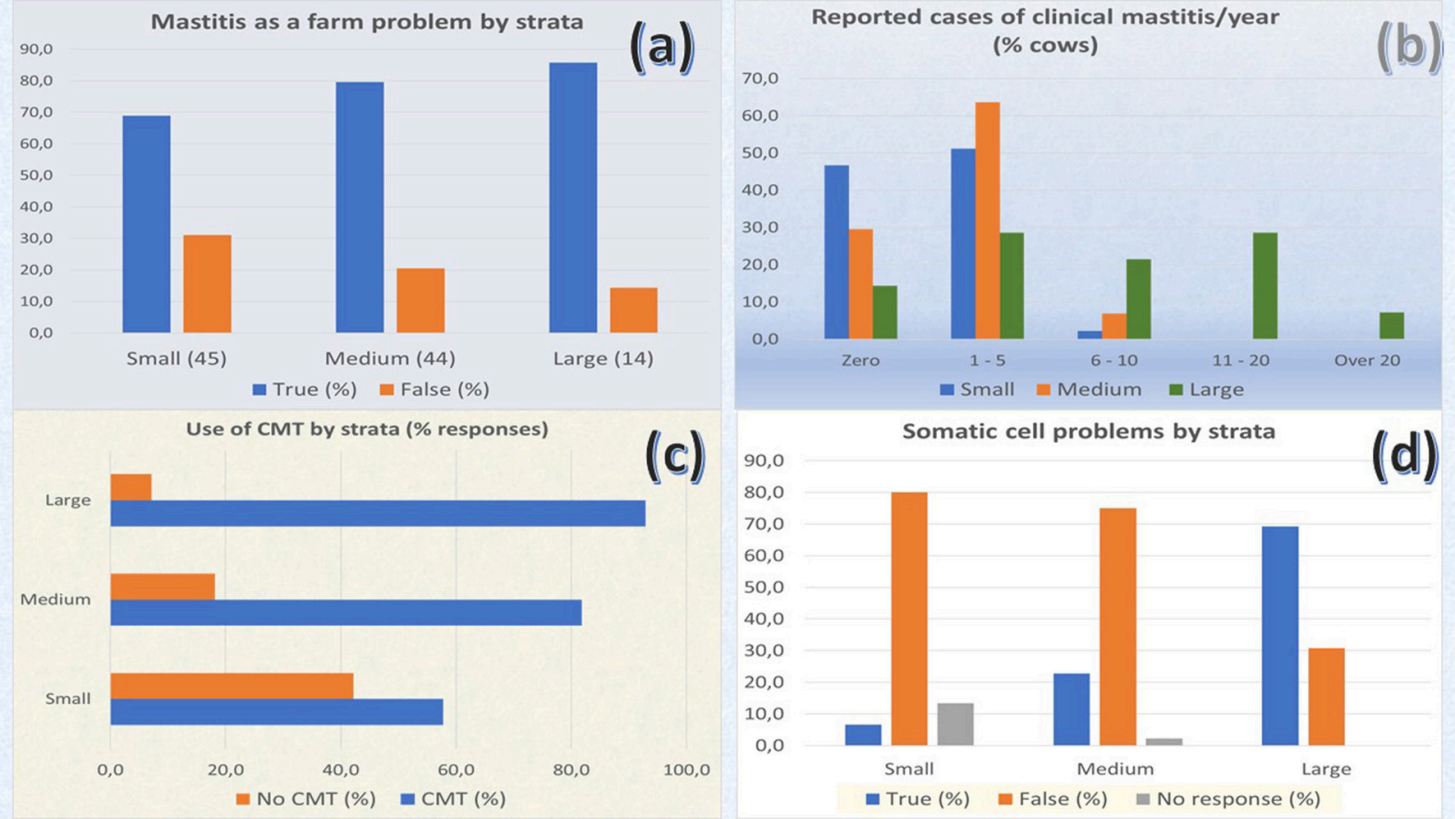
Producer perceptions about mastitis from questionnaire survey (n:103). Frequency of percentage of answers by farm strata **(a)** Mastitis as a problem in their farms. **(b)** Occurrence of clinical mastitis. **(c)** Use of CMT at farm. **(d)** Buyers complaining about SCC in milk.

According to the questionnaire survey (*n* = 103), mastitis was considered a problem by 68.9%, 79.5%, and 85.7% of the producers in small, medium, and large farms, respectively (Figure 2a); however, no significant differences were found in the proportions between strata (*X*^2^ = 2.254; *p* = 0.324). Despite the importance of the disease, producers reported a relatively low occurrence of cases of clinical mastitis per farm/year (Figure 2b).

Based on the participatory workshop (*n* = 55), producers were concerned about mastitis because of lower milk production (45 responses), animal health issues (18 replies), low milk quality (18 responses), public health concerns (12 responses), animal welfare (2 responses), increase in the rate of animal culling (3 responses), and other issues (4 responses).

According to the results of the questionnaire survey, producers appear not to be completely aware of the extent and impact of SCM on their production systems. In all strata, only 76% of the producers indicated SCM as a problem in their farms. However, CMT was a test routinely used for SCM detection at the farm level (Figure 2c). Large farms (92.9%) used CMT significantly more frequently than medium (82.9%) and small farms (58.1%) (*X*^2^ = 8.617; *p* = 0.0135) (n: 103).

Upon the question of whether producers have had any complaints about the SCC in the bulk tank from milk buyers, either middlemen or pasteurization plants, 6.7%, 22.7%, and 64.3% of the producers from small, medium, and large farms, respectively, reported having problems regarding SCC with milk buyers (*X*^2^ = 18.683; *p* < 0.01) (n: 103). Figure 2d.

The cross-sectional survey also indicated that, irrespective of the stratum, most of the producers tend to follow and apply a milking routine directed to reduce the impact of the disease (Table 3). Small producers used less mechanical milking systems and veterinary services than medium and large producers (*p* < 0.01). Medium and large producers exclusively used mechanical milking systems (100%).

**Table 3:** Number (percentage) and frequency of use of preventive measures for subclinical mastitis in farms participating in the cross-sectional study, arranged by strata (n: 55).

**Preventive measure**	**Small**	**Medium**	**Large**	***X*^2^ yates correction (*p*)**
CMT	15 (54%)	15 (71%)	6 (100%)	3.206 (0.201)
Washing udder	21 (75%)	15 (71%)	1 (17%)	5.349 (0.069)
Drying udder	21 (75%)	16 (76%)	3 (50%)	0.642 (0.725)
Sealing teats	17 (61%)	18(86%)	6 (100%)	3.923 (0.141)
Dry cow treatment	18 (64%)	15 (71%)	6 (100%)	1.606 (0.448)
Cleaning milk canteens	20 (71%)	19 (90%)	4 (67%)	1.651 (0.438)
Mechanical milking system	15 (54%)	21 (100%)	6 (100%)	12.88 (0.0016)
Veterinary services	20 (71%)	21 (100%)	6 (100%)	6.061 (0.048)
Total farms	28	21	6

### Financial losses and effects of preventive measures

The estimated financial milk losses due to SCM per farm were adjusted to 10-cow herd per farm per year to allow comparisons between strata (Table 4). The mean of financial milk losses (value of A10CML) per farm associated with the presence of SCM were US$ 692; the range of losses was wide in small and medium farm strata, and no differences were detected across strata (*F* = 1.703; *p* = 0.192).

**Table 4:** Descriptive statistics of yearly financial losses associated with SCM adjusted to 10 cows per farm per year stratum (US$).

**Farm strata**	***N***	**Mean**	**Min**	**Max**	**SE^♣^**
Small	28	572.3	0	3397.6	151.1
Medium	21	936.0	98.4	4601.0	174.5
Large	6	396.1	214.4	588.2	326.4

♣ *SE, Standard Error*.

Economic losses in the region were estimated for each stratum (Table 5). The economic impact of SCM due to milk losses in Area Five (11,619 cows) was estimated to be about US$800.000/year and $70.3 per cow/year. Despite the fact that small and large farm strata have higher region's share of farms and cows, respectively, the medium stratum contributes to the highest share of both milk and economic losses due to SCM.

**Table 5:** Regional and strata losses estimation per year, calculated for each stratum in Area Five, Bogota Plateau.

**Factor**	**Regional level**	**Proportion into strata**		
		**Small (%)**	**Medium (%)**	**Large (%)**
Farms	365	52	38	10
Cows	11,619	19	40	41
Estimated milk production (L)	43,714,447.8	19	47	34
Estimated milk losses due to SCM (L)	2,382,135.4	18	62	20
Estimated financial milk losses due to SCM (US$)	$816,361.5	17	62	21

Costs associated with the most frequent preventive and control measures for SCM reported on the questionnaire survey are shown in Tables 6, 7. Dry cow treatments were applied overall by 71% of the producers and included mainly two antibiotic choices: a mixture of cloxacillin 7% and ampicillin 3.5%^3^ and a combination of spiramycin and neomycin 5 g^4^. At the regional level, the costs associated with dry cow treatment varied from US $62,433 to US $107,149 (Table 6). Similarly, the costs associated with preventive milking routine practices depended on the type of sealant used (diluted iodine or a commercial product) and personnel (Table 7). On the other hand, the cost of pre-milking preparation of udders, locally referred to as “the milking routine,” depends mainly on labor costs and could rise to $191–288/month for a herd of 50 cows.

**Table 6:** Cost estimation (US $) of a single treatment of cows at drying off, calculated for the two veterinary drugs of more frequent use in the region and calculated regional cost of the conduct.

**Product**	**Quantity/cow**	**Cost lactation/cow**	**Total × 10 cows/year^♦^**
Secamil^Ⓡ^	4 syringes	$10.9	$113.1
Bovisec^Ⓡ^	4 syringes	$6.2	$65.9
Manpower	20 min	$0.4
**REGIONAL ESTIMATION**				
**Strata**	**Cows/region**	**Frequency of the conduct (% farms)**	**Lower cost**	**Higher cost**
Small	2,171	64	$9,156.4	$15,714.6
Medium	4,702	71	$22,000.2	$37,757.5
Large	4,746	100	$31,276.1	$53,677.3
Total	11,619		$62,432.7	$107,149.4

♦ *Includes manpower costs*.

**Table 7:** Cost estimation of a milking preventive routine using diluted iodine or a commercial product for sealing the udder (US $).

**Procedure**	**Product**	**Quantity/cow**	**Total cost/two milkings a day/10 cows/30 days**
Washing	Water	4 liters	$ 0.04
Drying off	Gazette paper	Two sheets	$ 0.93
Sealing	Iodine	10 ml	$ 0.21
	Sellodine^Ⓡ^	10 ml	$ 19.57
Personnel	Salary	3 min/milking (preventive routine)	$ 37.09
Total	Iodine		$ 38.27
	Sellodine^Ⓡ^		$ 57.63

The multiple ANOVA regression model used to predict financial losses from the use of disease management measures demonstrated that none of the preventive practices were individually associated with financial losses by SCM (*F* = 1.255; *p* = 0.291), although the use of the mechanical milking system had a significant effect on the model (*t* = 2.299; *p* = 0.026) (Table 8). Thus, a single regression model was performed to predict losses from the use of mechanical milking systems. This effect was nearly significant (*F* = 3.368; *p* = 0.072) indicating that this factor could be related with higher losses. The ANOVA model expost tests demonstrated that variances were heteroskedastic according to the White test (*F* = 4.04; *p* = 0.0002) and the absence of error autocorrelation according to the Breusch–Godfrey test (*F* = 1.057; *p* = 0.31). The uncentered variance inflation factors (VIF) shown in Table 8 revealed that only CMT use and dry cow treatment were not linearly associated (VIF value <5.0). As a consequence, the coefficients, although determined, show relatively high standard errors (29).

**Table 8:** Regression ANOVA model of financial losses (adjusted to 10 cows/year) for preventive measures of mastitis (n: 55).

**Source^♦^**	**Value**	**Error**	***t***	***p^♣^***	**Low CI 95%**	**High CI 95%**	**Uncentered VIF^♠^**
Constant	234.81	716.58	0.328	0.745	−1208.5	1678.1	43.141
CMT	−338.12	251.3	−1.346	0.185	−844.3	168.0	3.537
Washing udder	−27.85	312.35	−0.089	0.929	−656.9	601.3	5.616
Drying udder	208.14	340.31	0.612	0.544	−477.3	893.6	7.207
Sealing teats	−266.85	308.60	−0.865	0.392	−888.4	354.7	6.223
Dry cow treatment	−370.57	260.81	−1.421	0.162	−895.9	154.7	4.233
Cleaning milk canteens	121.83	453.79	0.268	0.790	−792.2	1035.8	16.019
Mechanical milking system	723.25	314.58	2.299	0.026*	89.7	1356.9	6.466
Veterinary services	378.84	522.59	0.725	0.472	−673.7	1431.4	21.669

♦ *Dependent variable: Adjusted loss (US $) to 10 cows/year*.

♣ ^*^Indicates a significant coefficient (p < 0.05).

♠ *VIF, Variance Inflation Factors; VIF> 5 - < 10 indicates moderate collinearity and VIF > 10 indicates severe collinearity*.

In addition to these ANOVA model expost tests results, the PCA was conducted in order to find ways of reducing multicolineality and heteroskedacity. The KMO measure of sampling adequacy was 0.529, which was considered acceptable, being higher than 0.504 (28). The values for the Bartlett sphericity test were significant (*X*^2^ = 62.913; d.f. = 28; *p* = 0.001) (28). Additionally, sequential process of PCA was followed, to group preventive measures into a reduced set of three variables, which are uncorrelated with each other and accounted for decreasing proportions of the total variance of the original variables using SPSS statistics 22^Ⓡ^ (28, 29).

The PCA of prevention control measures showed relevance of three first factors (Table 9). The eigenvalues associated with each component (factor) before extraction, after extraction, and after rotation identified eight linear components, of which the first three explained 60% of the data variance. These three factors followed Kaiser's rule (28), and the variance shared among them is shown in Table 9. Table 10 shows the communalities before and after extraction and the contribution of each intervention to the shared variance of the group. The orthogonal rotation of factors, following Varimax approach, identified the factor loading and the interventions (bold) that belong to each of the three factors (Table 11) (30).

**Table 9:** Total variance explained, extraction method: principal component analysis.

**C^♦^**	**Initial eigenvalues**			**Extraction sums of squared loadings**			**Rotation sums of squared loadings**		
	**Total**	***%S^2^***	**Σ %*S^2^***	**Total**	***%S^2^***	**Σ %*S^2^***	**Total**	***%S^2^***	**Σ %*S^2^***
1	1.985	24.82	24.82	1.985	24.82	24.82	1.737	21.71	21.71
2	1.742	21.77	46.59	1.742	21.77	46.59	1.686	21.08	42.79
3	1.090	13.62	60.21	1.090	13.62	60.21	1.393	17.42	60.21
4	0.887	11.09	71.29					
5	0.796	9.95	81.24					
6	0.698	8.72	89.96					
7	0.502	6.27	96.23					
8	0.301	3.77	100.00					

♦ *C, Component of variance—Management factors*.

**Table 10:** Communalities.

**Variables**	**Initial**	**Extraction**
CMT	1.000	0.447
Cleaning udder	1.000	0.755
Drying udder	1.000	0.784
Sealing teats	1.000	0.644
Dry cow treatment	1.000	0.512
Cleaning milk canteens	1.000	0.470
Mechanical milking system	1.000	0.676
Veterinary services	1.000	0.530

*Extraction method: Principal component analysis*.

**Table 11:** Rotated (Varimax) component matrix.

	**Loading factors scores**		
**Variables**	**1**	**2**	**3**
CMT	0.022	0.133	**0.845**
Cleaning udder	**0.984**	0.016	0.000
Drying udder	**0.991**	0.008	0.000
Sealing teats	0.001	**0.985**	0.014
Dry cow treatment	0.000	0.000	**1.000**
Cleaning milk canteens	0.018	0.004	**0.978**
Mechanical milking system	0.081	**0.867**	0.052
Veterinary services	0.272	**0.727**	0.001

*Factor loading—three factors*.

*Extraction method: Principal component analysis*.

*Rotation method: varimax with Kaiser Normalization*.

*Bold indicated factors scores have values over 0.400 and identify interventions that contributed to the matrix*.

The regression model of adjusted financial losses depending on the three factors is shown as follows:

Value of A10CML = 695.5 + 20.8 (Factor1) +163.6 (Factor2) - 191.3 (Factor3).

The *t* values and the corresponding probabilities for the factors were as follows: F1 (*t* = 0.189; *p* = 0.851), F2 (*t* = *1*.489; *p* = 0.143), and F3 (*t* = 1.741; *p* = 0.088) and the regression *R*^2^ = 0.096.

The model confidence is appropriate (White test: *F* = 0.429; *p* = 0.9126), and errors are not correlated according to the Breusch–Godfrey test (*F* = 0.172; *p* = 0.678).

## Discussion

### Financial losses

The methodological approach used in this study allowed us to estimate the prevalence of SCM and corroborate its relevance as an invisible problem that can cause financial losses to producers through the reduction of milk production (1, 3, 4, 12, 30). It is remarked that although epidemiological design was intended for a fully probabilistic sampling, as explained previously, field conditions led the authors to include farms following producer's willingness, resulting in a convenience sampling. However, it is considered that the sample is representative of the regional dairy, since a proportional number of farms of each stratum was included, according to the sampling fraction. Lack of data on variables such as intervention costs and performance prevented us from estimating other financial losses associated with SCM. Economic models that estimate milk losses caused by mastitis at both regional and local levels can be useful for implementing decision support systems that reduce the impact of the disease. In our study, the financial impact of SCM varied among farms irrespective of the stratum. Standardized adjusted yearly (305 days lactation length) milk losses for 10 cows/farm allowed comparing farms irrespective of the stratum and provided a regional picture. Therefore, there were no statistical differences in the values of A10CML between strata, contrasting with the prevalence of SCM in cows.

Nevertheless, financial losses were higher in the medium-sized farm strata, but variability, expressed by the SE, is high in all strata (Table 4). In general, it seems that large farms are more homogeneous in their management of SCM, and have lower financial losses despite productivity and better farm prices. Absolute farm values of both milk and financial losses depend on farm productivity, size, and market milk price.

Regional financial losses are high. They correspond to the reduction of regional milk supply, but individual farm losses are beyond the scope of the results of this research as individual production costs and gross margins were not calculated (1, 9). Despite the lack of information to calculate farm gross margin, it is assumed that measures to avoid milk losses will increase returns of producers to production costs, because the disease limits their efficiency and, therefore, the profitability (31). The main source of both losses and improvement opportunities is the farm. Therefore, the producer's decision-making is crucial, and further research is recommended to study the same.

Based on our findings, there is a larger room for improvement on the medium farm stratum. Despite such a regional financial impact, it seems like there is no incentive for the small farm producers to adopt changes, as the absolute value of estimated milk losses is low. Absence of any price incentive associated with SCC reinforces the lack of interest from producers to use diagnosis or implement control methods for SCM (32–34). Estimation of the financial impact could be used to advocate for the implementation of prevention methods that reduce the impact of SCM in Colombian dairies.

### SCM management

Although the advocacy of prevention measures by the use of economic impact assessments is quite important, the intervention efficiency is a cornerstone of economics in animal health (26, 33). Both the cross-sectional and questionnaire surveys indicated that most of the producers tend to follow and apply mastitis control management practices irrespective of the stratum. The study does not allow the evaluation of effectiveness or performance of specific prevention measures as no individual appraisal or follow-up measure was performed. The weak statistical association between preventive measures and milk economic losses due to SCM encourage us to conduct further research or provide a better understanding of SCM management (8–10, 34) through in-depth research on intervention performance, operational appraisals, and evaluation of intervention effectiveness (intervention cost vs. avoided losses).

Moreover, the high range of of the values of milk loss among farms and strata prevent any generalization in terms of efficiency and effectiveness of preventive measures for SCM implemented in each stratum. Therefore, the ANOVA expost tests, which result in multicollinearity and heteroskedacity, sustained the boundaries of this approach and the potentiality of the PCA (28, 29, 35–37). The PCA grouped measures into three components (factors). The first factor was related to the basic practices of milking hygiene. The second factor included activities that could be described as higher levels of hygiene and veterinary advice. The third factor was associated with activities related to diagnosis (CMT) and treatment of the dry cow as medical interventions. Additive effects of interventions of these factors would provide insights on how interventions work together, irrespective of measures of the individual effect of which significance was not found.

The estimation of intervention costs of the most common preventive measures indicates that the investments of producers against the disease are relevant considering the financial losses due to SCM. The intervention cost evaluation per farm was beyond the aims of this paper; therefore, a better understanding of intervention and its effect on the reduction of losses is needed so that the decision-making processes can be improved (3, 9, 34).

The value of SCC per quarter as predictor of losses and potential indicator of intervention effectiveness was demonstrated. At the field level in Colombia, the CMT is much more suitable than the Porta SCC^Ⓡ^ system because of test availability and costs. The CMT was used more frequently in the stratum of large farms, but it is necessary to understand the limitation of this test. Here, a result of grade 1 (slightly positive) corresponds to SCC between 400–1200 × 10^3^ cells/ml (23), which implies that this test could not detect quarters affected with SCM but that do not have a high SCC.

A phenomenon that appears to occur in the region is that the SCC requirements of the private pasteurization plants could be favoring a greater attention to SCM in large farms; for this reason, the producers in this stratum are trying to reduce the incidence of the disease and, therefore, its economic impact. In other strata, incentives are not present as they are used to selling their milk through informal market channels. Some pasteurization plants have established some price incentives for producers with low SCC. Therefore, producer's committees and pasteurization plants are providing technical advice and training on mastitis diagnosis management. Nevertheless, there are a lot of middlemen buying raw milk at farms who do not provide any service or incentive to improve milk quality. The government has set rules toward price incentives for raw milk quality according to the total solid content and CFU (<175,000–300,000) differentiating standards for specialized dairy and dual-purpose production^5^. These regulations do not include any aspect regarding SCC. This contrasts the rules in other countries such as the USA where the SCC for bulk tank milk grade A is 750 × 10^3^ cells/ml (32).

Results of this study and microbiological studies in milking areas of the country indicated that the most prevalent etiological agents of SCM are contagious organisms (19–21, 23). It would be expected that any intervention measure is based on an accurate diagnosis, however, the price of the diagnostic test is prohibitive. It is estimated that the price of the diagnostic test per quarter is equivalent to 25 liters of milk at farm price. The design of the prevention measures that excluded diagnosis could explain the lack of effectiveness of the preventive program shown above.

In this research, veterinary service is used and advice is apparently followed; however, a larger improvement room is feasible for both SCM detection and its management where high direct financial losses were found at both regional and farm levels. This apparent need for veterinary services in the dairy sector contrasts the low overall demand for veterinary services found in cattle production in Colombia (35). Further research on the assessment of the economic impact of SCM and effectiveness of intervention measures would improve our understanding of the disease (26, 27, 35). A major involvement of producers could also enhance their perception about the problem of SCM (9, 34, 37).

## Author contributions

CM: data management; econometric analysis: variance analysis, principal component analysis; EB: lead epidemiological data analysis and interpretation; leader of the field data collection and epidemiological studies; JR: lead economic analysis and modeling, paper framework, and scope; presenter of preliminary results at the ISSEAH meeting at Scotland.

### Conflict of interest statement

The authors declare that the research was conducted in the absence of any commercial or financial relationships that could be construed as a potential conflict of interest.

## Footnotes

^1^http://www.winepi.net

^2^http://epitools.ausvet.com.au/content.php?page=2StagePrevalence2.

^3^Secamil®; Virbac Colombia Ltd. Bogota, Colombia.

^4^Bovisec®; Genfar, S.A. Bogota, Colombia.

^5^Decree 616/2006 Ministry of Social Protection and Resolution 017/2012, Ministry of Agriculture.
